# Image segmentation and coverage estimation of deep-sea polymetallic nodules based on lightweight deep learning model

**DOI:** 10.1038/s41598-025-89952-8

**Published:** 2025-03-24

**Authors:** Yue Hao, Shijuan Yan, Gang Yang, Yiping Luo, Dalong Liu, Chunhua Han, Xiangwen Ren, Dewen Du

**Affiliations:** 1 Key Laboratory of Marine Geology and Metallogeny, First Institute of Oceanography, Ministry of Natural Resources, Qingdao, 266061 China; 2 Laboratory for Marine Mineral Resources, Qingdao Marine Science and Technology Center, Qingdao, 266237 China; 3 National Marine Data and Information Service, Tianjin, 300012 China; 4 Key Laboratory of Deep Sea Mineral Resource Development, Shandong(Preparatory), Qingdao, China

**Keywords:** Polymetallic nodule coverage, Deep learning, YOLOv7-PMN, MobileNetV3, Depth-wise separable convolution, Semantic segmentation, Ocean sciences, Computer science

## Abstract

Deep-sea polymetallic nodules, abundant in critical metal elements, are a vital strategic mineral resource. Accordingly, the prompt, accurate, and high-speed acquisition of parameters and distribution data for these nodules is crucial for the effective exploration, evaluation, and identification of valuable deposits. Studies show that one of the primary parameters for assessing polymetallic nodules is the Coverage Rate. For real-time, accurate, and efficient computation of this parameter, this article proposes a streamlined segmentation model named YOLOv7-PMN. This model is particularly designed for analyzing seafloor video data. The model substitutes the YOLOv7 backbone with the lightweight feature extraction framework of MobileNetV3-Small and integrates multi-level Squeeze-and-Excitation attention mechanisms. These changes enhance detection accuracy, speed up inference, and reduce the model’s overall size. The head network utilizes depth-wise separable convolution modules, significantly decreasing the number of model parameters. Compared to the original YOLOv7, the YOLOv7-PMN shows improved detection and segmentation performance for nodules of varying sizes. On the same dataset, the recall rate for nodules increases by 3% over the YOLOv7 model. Model parameters are cut by 61.78%, memory usage by the best weights is reduced by 61.15%, and inference speed for detection and segmentation rises to 65.79 FPS, surpassing the 25 FPS video capture rate. The model demonstrates strong generalization capabilities, lowering the requirements for video data quality and reducing dependency on extensive dataset annotations. In summary, YOLOv7-PMN is highly effective in processing seabed images of polymetallic nodules, which are characterized by varying target scales, complex environments, and diverse features. This model holds significant promise for practical application and broad adoption.

## Introduction

Polymetallic nodules are dark brown or black, spherical formations composed of iron and manganese oxides and hydroxides encasing a core^1^. These nodules contain numerous rare and valuable metal elements, including cobalt, nickel, manganese, copper, rare earth elements, molybdenum, and platinum group metals^2^. Typically measuring between 3 and 10 cm in diameter, they can be ellipsoidal or joined together and are found on deep-sea plains and basins at depths of 4000 to 6000 m^3–5^. The rising demand for scarce metals like cobalt and nickel, driven by global industrialization and advancements in new energy technologies, has highlighted the importance of polymetallic nodules as a strategic mineral resource^6^. China is currently exploring these resources, with various stages of prospecting underway in its contracted exploration areas. Rapidly obtaining continuous and high-precision data on nodule deposit resource parameters and understanding their distribution is essential for exploring, evaluating, and defining rich polymetallic nodule deposits.

The coverage rate (CR) of polymetallic nodules indicates the proportion of the seafloor covered by these nodules per unit area. This parameter is crucial for assessing deep-sea polymetallic nodule reserves and evaluating their resource status. Traditionally, the coverage rate is calculated using the grid counting method, a statistical approach that determines the percentage of the area covered by nodules collected by a box sampler relative to the sampler’s opening area. This method depends largely on geological sampling to gather nodule resource data. However, in recent years, survey methods using underwater imaging technology have gained prominence^7,8^. These methods employ underwater cameras or photographic equipment to continuously capture high-resolution images of the seafloor, which are then analyzed to detect nodules and calculate their coverage as a percentage of the total area^9^. Compared to geological sampling, near-seabed imaging offers benefits such as greater efficiency, continuity, and visual clarity^10^.

Estimating coverage from video or photographic data relies on advanced computer vision and image analysis techniques. In this context, Dong et al.^11^ applied an enhanced Mask R-CNN network to various nodule image sets, taking into account different regions, light conditions, abundances, depths, and angles. Their model achieved an 85.575% recall rate, surpassing the performance of U-Net^12^, improved U-Net^13^, and Conditional Generative Adversarial Network (CGAN)^14^ models trained on the same dataset. Song et al.^15^ used the Pix2PixHD algorithm, derived from CGAN, to segment 106 clear nodule images, resulting in a recall rate of 98.55%. Wang et al.^16^ incorporated pyramid upsampling and residual modules into a U-Net model, achieving a 99.66% detection accuracy on 49 annotated images. Shao et al.^17^ developed a two-stage model using the Denoising Diffusion Probabilistic Model (DDPM) for semantic segmentation of seafloor images, achieving an 86.49% recall rate on a dataset of 106 annotated images and effectively handling poor lighting and high-density conditions. Liu et al.^18^ integrated data augmentation with an enhanced U-Net model and trained it on 64 images obtained under three different turbidity levels in pool environments, achieving a detection accuracy of over 90% across varying turbidity conditions. Schoening et al.^19^ introduced Compact Morphology-based Nodule Delineation (CoMoNoD), a two-stage image processing algorithm that detected nodules in 34,200 seafloor photographs in 19 h at a rate of 0.5 images per second. Tomczak et al.^20^ proposed a U-Net-based framework for detecting polymetallic nodules in 30,000 photographs in approximately 10 h, with a processing rate of 0.83 images per second.

The You Only Look Once (YOLO) family of object detection^21^ algorithms has been extended to instance^22^ and semantic segmentation^23^ models and applied across various fields, including agriculture, transportation, mining, medicine, and industry. The YOLO series has evolved from high-speed, low-accuracy models to high-speed, high-accuracy ones, enhancing practicality and reliability. Redmon et al.^24^ introduced the YOLO and Fast YOLO models, which processed images at 45 frames per second (FPS) and 155 FPS, respectively, with Mean Average Precision (MAP) scores of 63.4% and 52.7% on the PASCAL VOC dataset for object detection tasks, outperforming other real-time detectors of the time in both speed and accuracy. Li et al.^25^ compared Faster R-CNN, YOLOv3, and Single Shot Multi-Box Detector (SSD)^26^ for detecting greenhouses in high-resolution satellite images, demonstrating that YOLOv3 outperformed the others in both speed (73 FPS) and accuracy (93.2%). Soeb et al.^27^ developed a leaf disease image dataset and validated YOLOv7’s detection results, achieving a recall rate of 96.4%. Xia et al.^28^ integrated MobileNeXt and a dual-layer routing attention mechanism into YOLOv7, achieving an 89.9% recall rate on a tea disease image dataset. Albahli et al.^29^ proposed a melanoma localization and segmentation method based on YOLOv4^30^ and active contour segmentation, outperforming the top ten entries in the ISBI 2016 melanoma segmentation challenge. In seafloor mineral detection, Quintana et al.^31^ trained Fast-RCNN^32^ and DarkNet Yolo^24^ on a dataset of 6,823 nodules, with Fast-RCNN^32^ achieving a True Positive Fraction of 0.94 and DarkNet Yolo^24^ achieving 0.69. Sun et al.^33^ improved the YOLOv5s base network with Intersection over Union (IoU)^34^ and Wasserstein distance from optimal transport theory, training the model on a hyperspectral image dataset of 2,508 polymetallic nodules and achieving an accuracy of 84.5%.

Lightweight networks are designed to minimize network size and enhance speed while maintaining accuracy. Notable examples include the SqueezeNet^35,36^, ShuffleNet^37,38^, MnasNet^39^, and MobileNet^40–42^ series. Howard et al.^42^ developed MobileNetV1 using depth-wise separable convolutions, resulting in a model with about 4.2 million parameters, just 14.33% of a standard convolutional model, yet achieving comparable accuracy on the ImageNet dataset. Sandler et al.^43^ introduced MobileNetV2, which uses inverted residuals with linear bottlenecks, improving accuracy and speed with fewer layers, and having around 3.4 million parameters, fewer than MobileNetV1, ShuffleNet, and NasNet. Howard et al.^40^ proposed MobileNetV3-Large and MobileNetV3-Small, optimized using AutoML technology. It is worth noting that both models surpassed the performance of the SqueezeNet^35,36^, ShuffleNet^37,38^, and MnasNet^39^ networks on the ImageNet dataset with the same number of Multiply Accumulate Operations (MAdds). Specifically, MobileNetV3-Small reduced parameters to approximately 2.5 million. Lu et al.^44^ enhanced the YOLOv7 model for detecting steel strip surface defects by integrating MobileNetV3, depth-wise separable convolutions, and parameter-free attention mechanisms, reducing weight parameters by 20.7%.

Deep-sea polymetallic nodule surveys using video or photographic data encounter several challenges due to the harsh detection conditions and environmental factors^45,46^. The small and varied sizes of nodules and their diverse spatial distribution result in image data of varying quality^19^. These conditions create complex scenarios with multi-scale nodule images, diverse environmental influences, and varied target features^47^. Models that lack accuracy, are hard to deploy, have long inference times and weak generalization capabilities do not meet requirements in practical applications. For deep-sea polymetallic nodule detection, models must be highly efficient and accurate, ensuring detection precision while meeting real-time speed requirements. This study focuses on constructing a lightweight semantic segmentation model for polymetallic nodules, named YOLOv7-PMN (YOLOv7 for Polymetallic Nodules), based on near-seabed image data and considering the characteristics of nodule images. The main objective is to develop a lightweight model that addresses issues of low detection accuracy and slow speed in images of varying quality and scales. The research aims to optimize hardware resource usage, ease deployment, and enhance efficiency and accuracy. By addressing these challenges and refining the model, deep learning techniques can be innovatively applied to exploring deep-sea polymetallic nodules and improve the efficiency and accuracy of polymetallic nodule resource evaluation.

## Methods

### Deep learning networks

Deep learning network models, also known as Deep Neural Networks (DNNs), are advanced computational models inspired by biological neural networks. These models can learn intricate patterns from large datasets through multiple layers of neural nodes, enabling them to perform various tasks. For instance, Recurrent Neural Networks (RNNs)^48^ are used for text, speech, and time series data processing, while Convolutional Neural Networks (CNNs)^49^ are designed to process grid-like data structures such as images. The core principle behind these deep learning models involves optimizing model parameters using the gradient descent algorithm and calculating gradients through the backpropagation algorithm.

CNNs are particularly effective in image detection, natural language processing, and speech recognition. Their fundamental concept revolves around convolution operations to extract features from images, followed by multi-layer network structures for feature learning and classification. A typical CNN consists of input layers, convolutional layers, pooling layers, fully connected layers, and output layers. Input layers receive the raw image data. Convolutional layers apply multiple convolutional kernels to the input image, performing sliding operations to extract local features. The size, stride, and padding of the kernels determine the feature extraction range and precision. Different kernels can capture various features such as edges, textures, and shapes. Pooling layers downsample the output from the convolutional layer, reducing data volume and computational cost while enhancing the model’s robustness and tolerance to minor image variations. A fully connected layer flattens the feature vector from the pooling layer to learn global features. Finally, an output layer produces classification results or regression values based on the specific task. Additionally, activation functions link the outputs of upper-layer nodes to the inputs of lower-layer nodes, boosting the model’s expressive capability. Loss functions measure the discrepancy between the model’s predictions and the true values, guiding the training process. Optimizers adjust the model parameters to improve performance by minimizing the loss function.

#### YOLOv7 network

The YOLO series is a prominent collection of CNN algorithms, widely known for their efficiency and real-time object detection capabilities. Currently, these algorithms are widely used in the field of computer vision due to their ability to swiftly and accurately identify objects within images. Among various versions of the YOLO algorithm, YOLOv7^50^ exhibits a significant advancement. In the public COCO object detection dataset, YOLOv7 surpasses its predecessors and other models within the series in both accuracy and inference speed^50^.

**Fig. 1:**
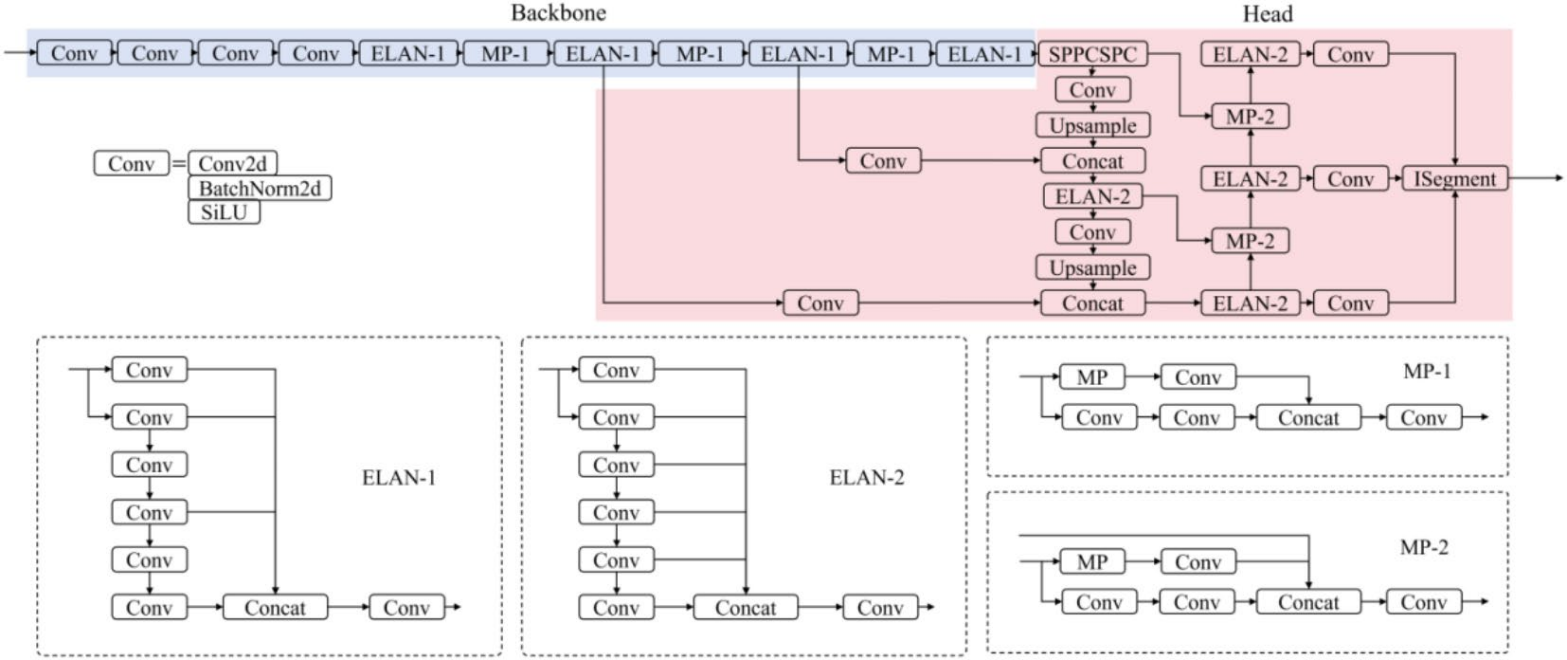
Structure of YOLOv7 semantic segmentation network.

As shown in Fig. 1, the YOLOv7 semantic segmentation network^51^ is structured with three primary components: a backbone network, a head network, and an ISegment module. The backbone network focuses on extracting feature information from the input image, while the head network utilizes the features provided by the backbone to perform semantic segmentation tasks. Moreover, YOLOv7 employs K-Means clustering to automatically generate anchor boxes for object detection and optimize the alignment of shape features across different target scales. It should be indicated that both the backbone and head networks incorporate Extended Efficient Layer Aggregation Network (ELAN) modules to adaptively aggregate features at varying scales. The ELAN-1 module aggregates features across four levels, while the ELAN-2 module does so across six levels, thus enhancing the preservation and extraction of feature information at different scales. The SPPCSPC module within the head network facilitates multi-scale feature fusion, improving the model’s ability to recognize targets of different sizes. Cross-scale feature map connections are established through upsampling (Upsample Module) and downsampling (MP-2 Module), enabling efficient information transfer between different resolution layers. A concatenation (Concat Module) operation merges features from various scales, creating richer and more comprehensive multi-scale representations. The ISegment module establishes independent segmentation heads for each scale of feature maps, allowing the segmentation of targets of diverse sizes. The integration between the backbone and head networks is inspired by the Feature Pyramid Network (FPN) structure^52^, which outputs feature maps at three distinct scales: large-scale for small targets, medium-scale for medium targets, and small-scale for large targets. This structure supports the model’s ability to independently predict segmentation at each feature map layer.

#### MobileNetV3 network

**Fig. 2:**
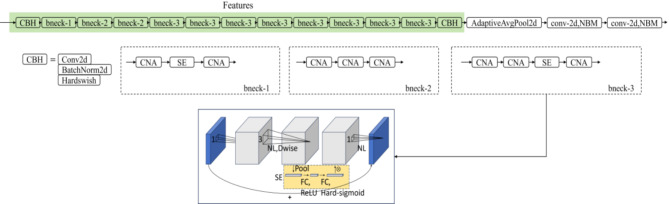
Structure of the MobileNetV3-Small network.

MobileNet lightweight algorithms are efficient convolutional neural network models designed specifically for mobile devices and embedded systems. MobileNetV3, a member of this series, integrates several advanced techniques: (1) Depth-wise Separable Convolution (DSC) from MobileNetV1; (2) Inverted Residual with Linear Bottleneck (IR) from MobileNetV2; (3) Squeeze-and-Excitation (SE) attention mechanism from MnasNet; (4) Hard-swish, a new activation function replacing the swish function; MobileNetV3 offers two variations: Large and Small. Figure 2 shows the structure of the MobileNetV3-Small network. The Small model is designed for scenarios with limited resources, requiring fewer parameters, reduced computational load, and faster processing speeds.

The ‘bneck’ module is the core modulus of MobileNetV3-Small, representing a variant of the IR structure. The feature extraction framework employs 11 bnecks of three types: (1) bneck-1 lacks the initial 1 × 1 convolution expansion layer; (2) bneck-2 does not include the SE module; (3) bneck-3 is the complete bneck module, incorporating SE, NL (Non-Linearity activation function), depth-wise separable convolution (Dwise), and IR. The bneck module works by expanding the feature space with a 1 × 1 convolution to increase channel numbers. It also uses a 3 × 3 depth-wise separable convolution to extract and integrate spatial and channel features, thereby reducing parameters and computations. Moreover, it enhances important features through the SE module and compresses features by reducing channel numbers using a 1 × 1 convolution.

The SE module consists of a global average pooling layer, two fully connected layers, and activation functions (ReLU and Hard-sigmoid). It compresses each channel’s feature map using global average pooling, generates importance weights for each channel through two fully connected layers, multiplies each channel’s importance weight with the original feature map, and adds the adjusted feature map back to the original to preserve information. The residual connection in the bneck module is conditional on having equal input and output channel numbers and a stride of 1. When these conditions are met, the input is directly added to the output, helping to mitigate the vanishing gradient problem.

In summary, MobileNetV3-Small optimizes network structure and parameters, significantly reducing computational complexity and model size while maintaining high accuracy.

#### Depth-wise separable convolution

**Fig. 3:**
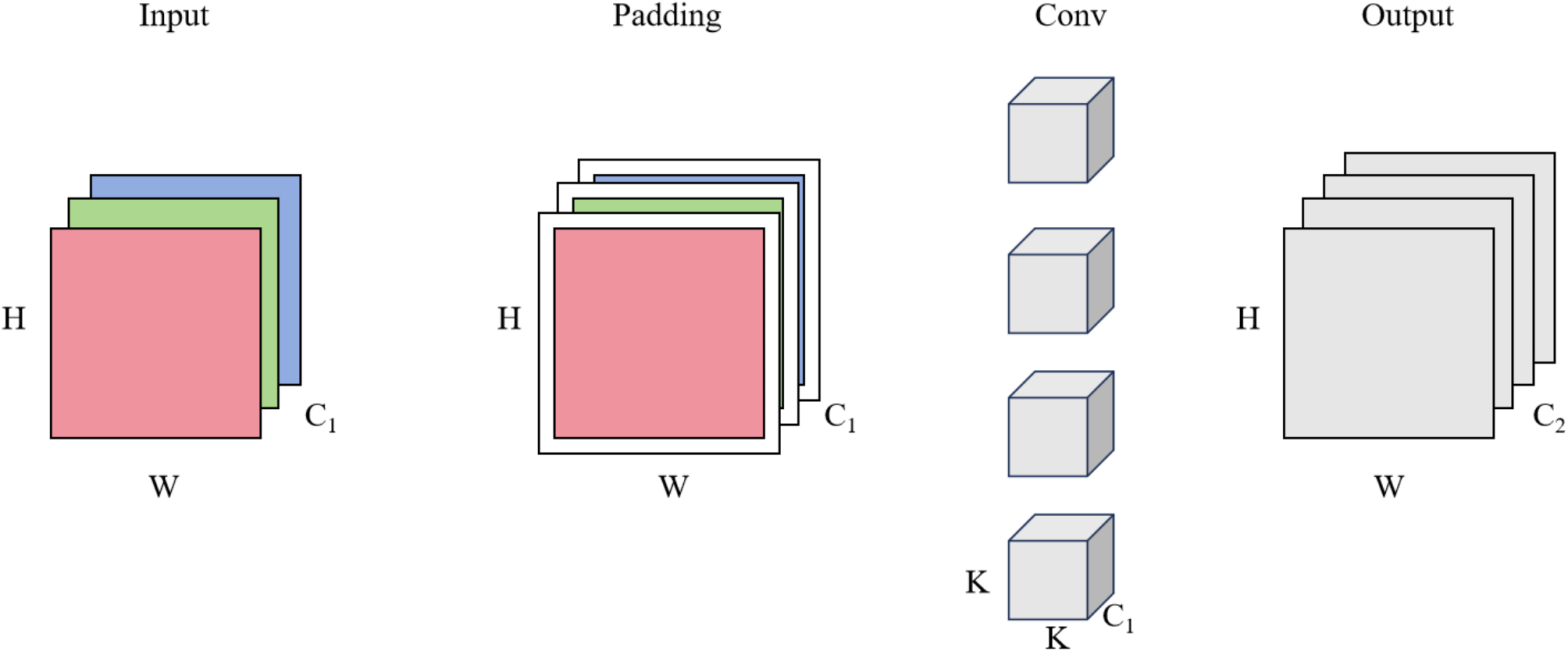
Structure of standard convolution with the same paddings.

Figure 3, Standard Convolution (SC) comprises input layers, padding layers, convolutional layers, and output layers. In this process, a convolution kernel or filter (of size K) traverses the input data by sliding from left to right and top to bottom, advancing by a stride S at each step. At every position, it performs dot product calculations to extract features from the windowed segment of the input data. The total number of convolution kernels used determines the number of channels in the final output image. These kernels process all input channels concurrently, thereby learning and capturing features across both the channels and spatial dimensions, which results in the generation of output feature maps directly from the input layer.

**Fig. 4:**
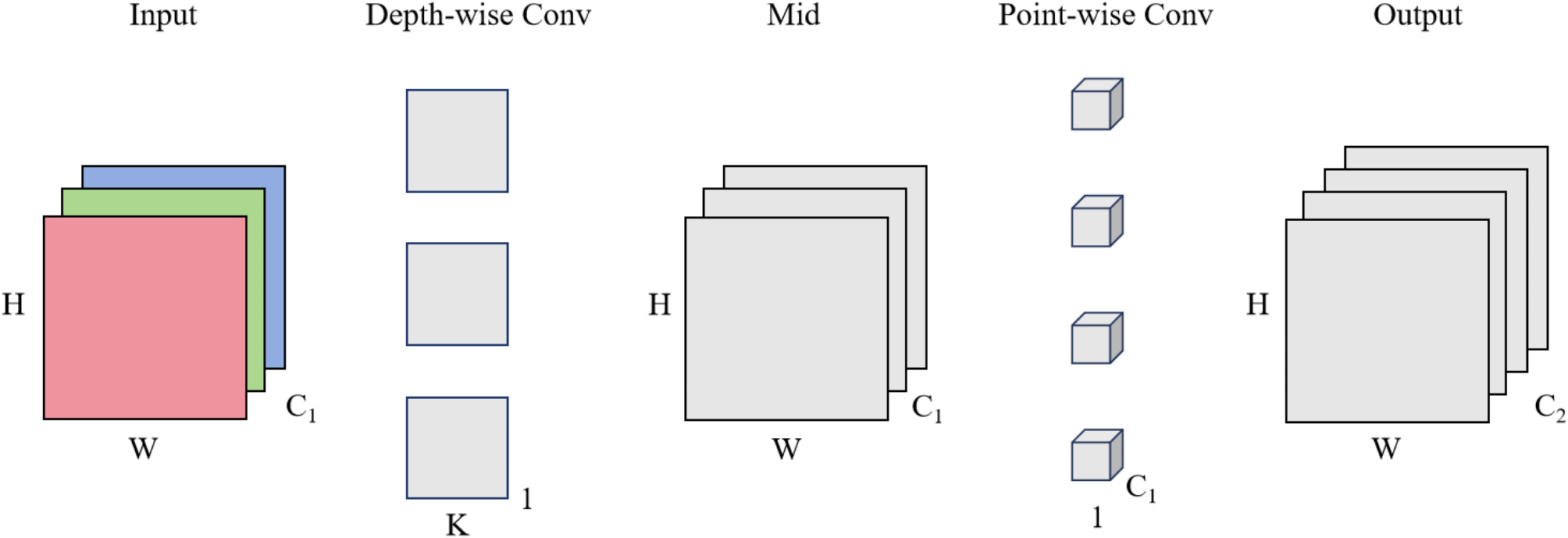
Structure of depth-wise separable convolution with the same Paddings.

Figure 4, Depth-wise Separable Convolution (DSC) consists of input layers, depth-wise convolution, intermediate layers, point-wise convolution, and output layers. This method simplifies the standard convolution process by splitting it into two distinct operations: depth-wise convolution and point-wise convolution. Depth-wise convolution applies K×K convolution kernels individually to each input channel, which focuses on learning spatial features. On the other hand, point-wise convolution uses 1 × 1 convolution kernels across all input channels at once, enabling the processing of combined channel information to learn and extract features specific to each channel. By separating the convolution process into these two stages, depth-wise separable convolution significantly reduces the computational complexity and the number of parameters needed, compared to the standard convolution.

The parameter count in standard convolution encompasses the weights of the convolution kernels and any bias terms involved in the computation. These parameters are directly linked to the number of input channels, output channels, and the dimensions of the convolution kernel. Figure 3 shows an image characterized by a height of H pixels, a width of W pixels, and C_1_ channels as the input data. Using the Same Padding, zeros are added around the image to maintain consistent output dimensions with the input dimensions post-convolution. Excluding the bias term, the formula for calculating the number of parameters in standard convolution is:

1 $${\text{Parameters }} = {\text{ K }} \times {\text{ K }} \times {\text{ C}}_{{{1}}} \times {\text{ C}}_{{{2}}}$$

In depth-wise separable convolution, the number of parameters in the depth-wise convolution is K×K×C_1_. Moreover, the point-wise convolution employs 1 × 1×C_1_ convolution kernels with C_2_ kernels (equal to the number of output channels). Therefore, the number of parameters is C_1_×C_2_. The number of total parameters is:

2 $${\text{Parameters }} = {\text{ K }} \times {\text{ K }} \times {\text{ C}}_{{{1}}} + {\text{ C}}_{{{1}}} \times {\text{ C}}_{{{2}}}$$

The ratio of parameters between depth-wise separable convolution and standard convolution is defined as follows:

3 $$\:\text{R}=\frac{\text{K}\:\times\:\:\text{K}\:\times\:\:{\text{C}}_{1}\:+\:{\text{C}}_{1}\:\times\:\:{\text{C}}_{2}}{\text{K}\:\times\:\:\text{K}\:\times\:\:{\text{C}}_{1}\:\times\:\:{\text{C}}_{2}}=\frac{1}{{\text{C}}_{2}}+\frac{1}{\text{K}\times\:\text{K}}$$

This parameter reduction highlights the efficiency of depth-wise separable convolution compared to standard convolution: (1) The ratio of parameters is always less than 1, indicating that depth-wise separable convolution consistently requires fewer parameters than standard convolution; (2) As the number of output channels C_2_ increases, this ratio decreases, indicating a more substantial reduction in parameters with a higher number of output channels; (3) Similarly, as the kernel size K increases, the ratio also decreases, demonstrating larger kernel sizes benefit more from the parameter reduction.

Overall, depth-wise separable convolution can significantly reduce the number of parameters in the model.

### Model improvements

This study employs the YOLOv7 semantic segmentation model as the baseline and introduces three key improvements:

1. Backbone replacement: The backbone network of the YOLOv7 model is replaced with the MobileNetV3-Small feature extraction structure, resulting in the new YOLOv7-MobileNetV3 model.
2. Convolution module substitution: The standard convolution module (Conv) in the YOLOv7 model is substituted with depth-wise separable convolution, leading to the creation of the YOLOv7-DSC model.
3. Combined enhancements: Both the backbone network is replaced with the MobileNetV3-Small feature extraction structure and the standard convolution module (Conv) in the head network is substituted with depth-wise separable convolution, forming the YOLOv7-PMN model.

#### YOLOv7-MobileNetV3

**Fig. 5:**
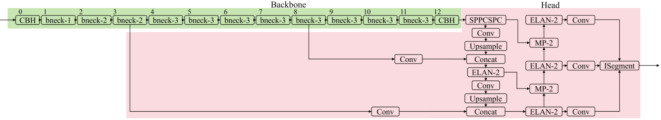
Structure of the YOLOv7-MobileNetV3 network.

In the YOLOv7-MobileNetV3 model, the original head network of the YOLOv7 semantic segmentation model is preserved, while the MobileNetV3-Small feature extraction structure (Features) is introduced as the new backbone network. The original backbone network of YOLOv7 feeds 8×, 16×, and 32× downsampled images into the head network. Thus, the new backbone network, MobileNetV3-Small, is connected to the existing head network at equivalent stages, specifically after the 8×, 16×, and 32× downsampling steps within the Features structure. These correspond to the 3rd, 8th, and 12th layers of the new backbone network, as depicted in Fig. 5.

### YOLOv7-DSC

**Fig. 6:**
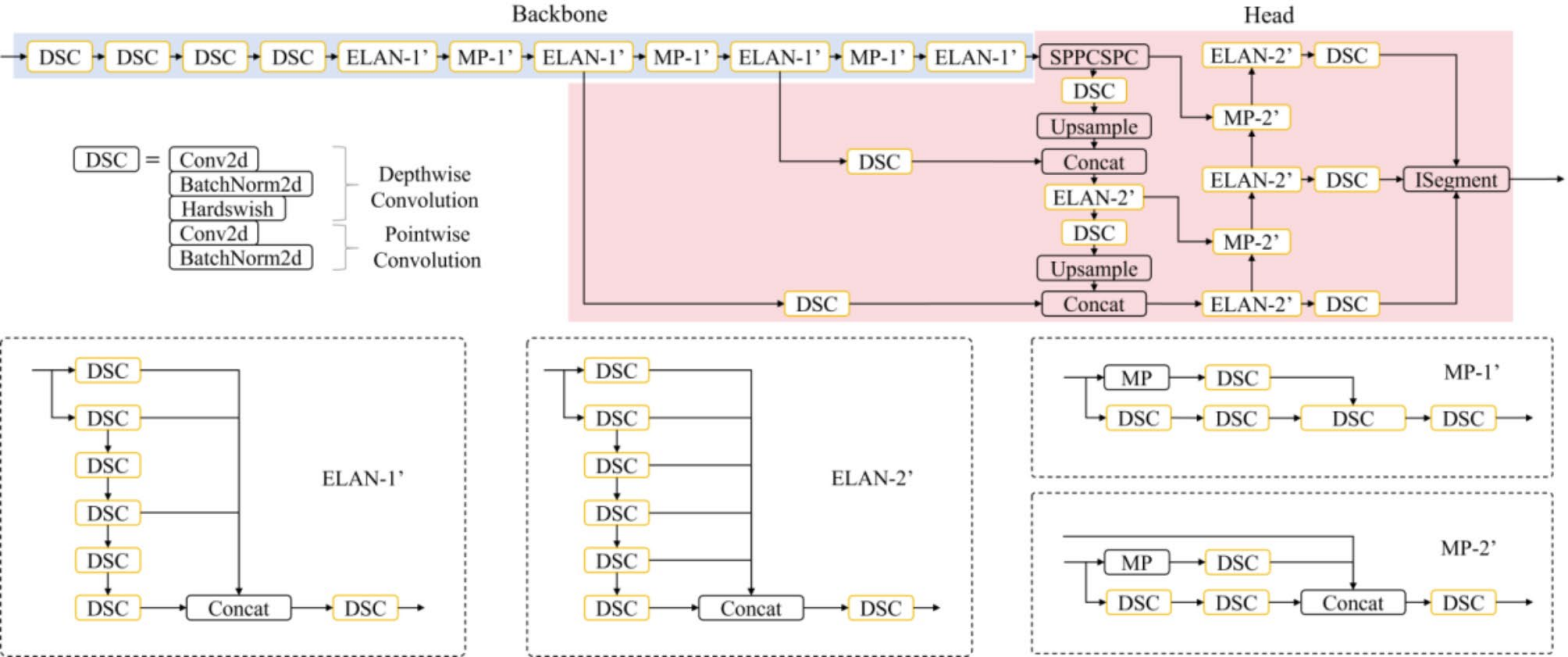
Structure of the YOLOv7-DSC network.

Figure 6 depicts the YOLOv7-DSC model, which incorporates depth-wise separable convolution in place of the standard convolution (Conv module shown in Fig. 1) used in the YOLOv7 semantic segmentation model. In this adaptation, all standard convolution Conv modules present within ELAN-1, ELAN-2, MP-1, and MP-2 of the YOLOv7 architecture, along with the standalone standard convolution Conv modules, are replaced with depth-wise separable convolution.

#### YOLOv7-PMN

**Fig. 7:**
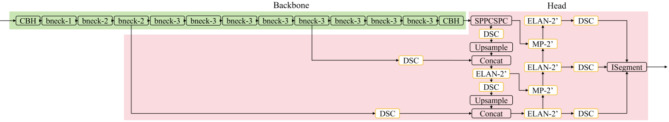
Structure of YOLOv7-PMN network.

Figure 7 illustrates the structure of the YOLOv7-PMN model. Compared to the YOLOv7 semantic segmentation model, the YOLOv7-PMN model retains the connections between the backbone network and the head network, as well as the intra-module connections within the YOLOv7 head network. This retention supports the effective transfer of multi-scale feature information. The model employs SPPCSPC, Upsample, and Concat modules for feature transmission and fusion, while the ISegment head is responsible for detecting and segmenting objects of different sizes in the image. The model integrates the MobileNetV3-Small feature extraction framework (Features) as the backbone network and replaces the standard convolution Conv in the network of the model header with depth-wise separable convolution. By merging the feature extraction structure with depth-wise separable convolution, this new model improves multi-scale object feature extraction, thereby boosting detection and segmentation precision. Meanwhile, it reduces the number of model parameters and computational complexity, resulting in lower weight memory usage and faster inference performance.

## Model application and results

### Dataset building

The dataset utilized in this research is primarily sourced from the Chinese Ocean Polymetallic Nodule Survey, conducted in the Clarion-Clipperton Fracture Zone (CCZ) of the equatorial northeastern Pacific. This dataset encompasses both positioning data for polymetallic nodules and associated image data.

#### Positioning data

The near-bottom image data used in this research were positioned using the POSIDONIA II ultra-short baseline (USBL) positioning system. This system, developed by IXBLUE Company, is a high-precision and long-distance ultra-short baseline underwater positioning system. It offers a maximum positioning depth of 6000 m, exceeding the average water depth in the region, and can achieve a maximum slant range of up to 10,000 m. Due to the variability in on-site measurement conditions and equipment performance, the underwater positioning data can be inconsistent, leading to potential inaccuracies. Consequently, the USBL data exhibited some degree of discretization, necessitating processing. To address this issue, a four-dimensional filtering approach was applied, incorporating the three-dimensional parameters (longitude, latitude, and water depth) along with time. This method effectively eliminated the severe jumping points and enhanced the continuity and stability of positioning data^53^.

#### Image data

The video data utilized in this study was captured with a high-resolution camera, achieving a resolution of 1,920 × 1,080 pixels. The camera was positioned about 7–9 m above the seabed, providing an average coverage of around 30 square meters per image. When the images were cropped to dimensions of 512 × 512, the coverage area per cropped image averaged 3.8 square meters, corresponding to an actual seafloor size of roughly 2 m × 2 m. During data collection, the carrier platform typically moves at a speed varying from 1 to 2 knots (where 1 knot equals 1.852 km/hour). The camera lens on the collection equipment is usually aligned perpendicularly to the seabed. Variations in the platform’s speed can cause polymetallic nodules in the images to appear stretched or elongated. Additionally, the distance between the platform and the seabed fluctuates even under stable conditions.

Simple statistics indicate that with an average platform speed of 0.373 m/s (0.725 knots), the distance fluctuation period ranges from about 70 to 740 s, with an average of approximately 6.8 min. This fluctuation affects light absorption and scattering, altering the light intensity in various wavelengths entering the camera lens and ultimately changing the total pixel values in each channel of the visible image. Based on the total pixel values of each channel, images are classified as near-distance, mid-distance, and far-distance. The classification process for a single image involves the following steps: (1) Calculate the total pixel value for the red, green, and blue channels of the visible image, denoted as R, G, and B, respectively; (2) If R $\:\ge\:$ B, meaning the total pixel value of the red channel is not less than the blue channel’s, the image is categorized as a near-distance image. Otherwise, the image is considered mid-distance or far-distance, requiring further evaluation; (3) If G $\:\ge\:$ B, indicating the total pixel value of the green channel is not less than the blue channel’s, the image is classified as mid-distance. Otherwise, it is identified as a far-distance image.

**Fig. 8:**
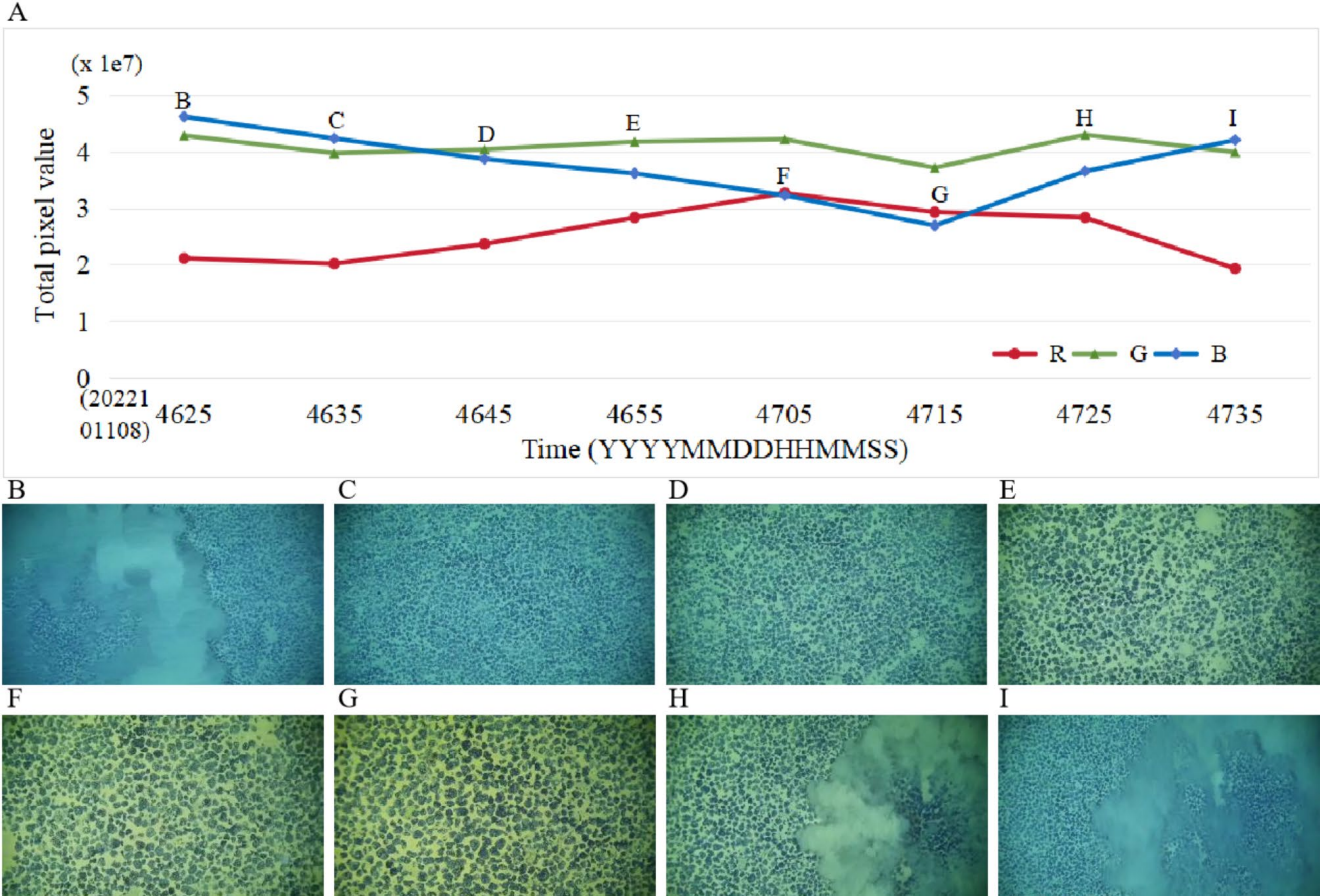
Image Data Classification.

In Fig. 8A, the horizontal axis represents the time series, and the vertical axis indicates the total pixel value for each channel. As shown in Fig. 8B-I, the camera approaches the seabed, and the image color shifts from deep blue to deep green and then to dark yellow, resulting in larger and fewer nodules in the image. Conversely, as the camera moves away from the seabed, the color transition reverses, leading to smaller and more numerous nodules in the image. This variability in nodule size and features is further influenced by water visibility, sediment color, biological organisms, bioturbation, and other seabed materials.

Figure 8B, C,I are classified as far-distance images, Fig. 8D, E,H as mid-distance images, and Fig. 8F, G as near-distance images. These eight images were sampled at 10-second intervals from a randomly selected video recorded by AUV during a 70-second motion period. At 08:46:25, when the AUV was far from the seabed, a significant amount of blue light reached the camera sensor, resulting in a high total pixel value for the blue channel (Fig. 8B). At 08:46:45, as the AUV moved closer to the seabed, green light attenuation was diminished, allowing more green light to reach the camera sensor and causing the total pixel value of the green channel to increase, surpassing the blue channel (Fig. 8D). At 08:47:05, the AUV was even closer to the seabed, enabling a large amount of red light to reach the sensor, which led to the red channel’s total pixel value exceeding that of the blue channel (Fig. 8F).

**Fig. 9:**
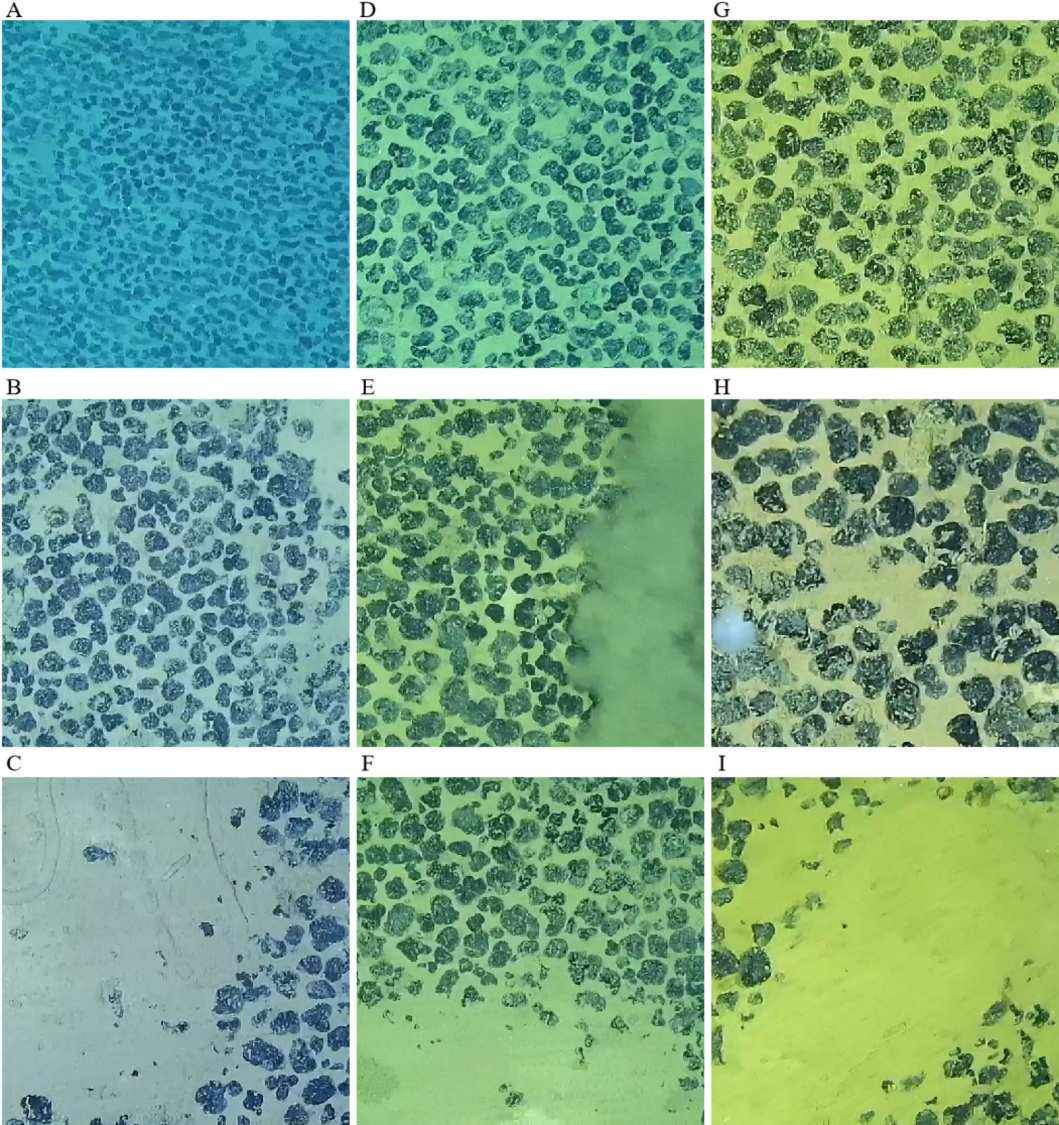
Dataset. (**A-C**), far-distance images; (**D-F**), mid-distance images; (**G-I**), near-distance images. (**E**), image with suspended particle obstruction; (**H**) and (**C**), images with biological obstruction or traces; (**C**), (**F**), and (**I**), images with sediment coverage on nodules.

A polymetallic nodule dataset is screened and constructed based on the distance at which the images are captured, as well as various image features such as clarity, brightness, contrast, color, number and shape of nodules, and the presence of resuspended particles, biological entities, or obstacles. As illustrated in Fig. 9A-I, the dataset exhibits the following characteristics: (1) Far-distance images: Captured from a higher altitude above the seafloor, these images cover a larger area and appear bluish, making the nodules look smaller. Each image may contain between 550 and 1,000 nodules. (2) Mid-distance images: These are taken from a moderate height, resulting in a greenish tint and medium-sized nodules. Each image typically features 200 to 550 nodules. (3) Near-distance images: These are captured very close to the seafloor, covering a smaller area and appearing yellowish. Nodules appear larger, with each image containing between 30 and 200 nodules. There are also special cases in the images. Suspended Particle Obstruction (Fig. 9E) means that sediment particles stirred up by the equipment partially obscure the nodules. Biological Obstruction (Fig. 9H, C) shows marine life of various shapes, colors, and sizes on the seafloor. Larger organisms may block one or more nodules, while smaller organisms may appear as multiple tiny white spots on a single nodule. Sediment Coverage refers to nodules partially or fully covered by sediment, making their boundaries incomplete or unclear in the images (Fig. 9C, F,I).

**Fig. 10:**
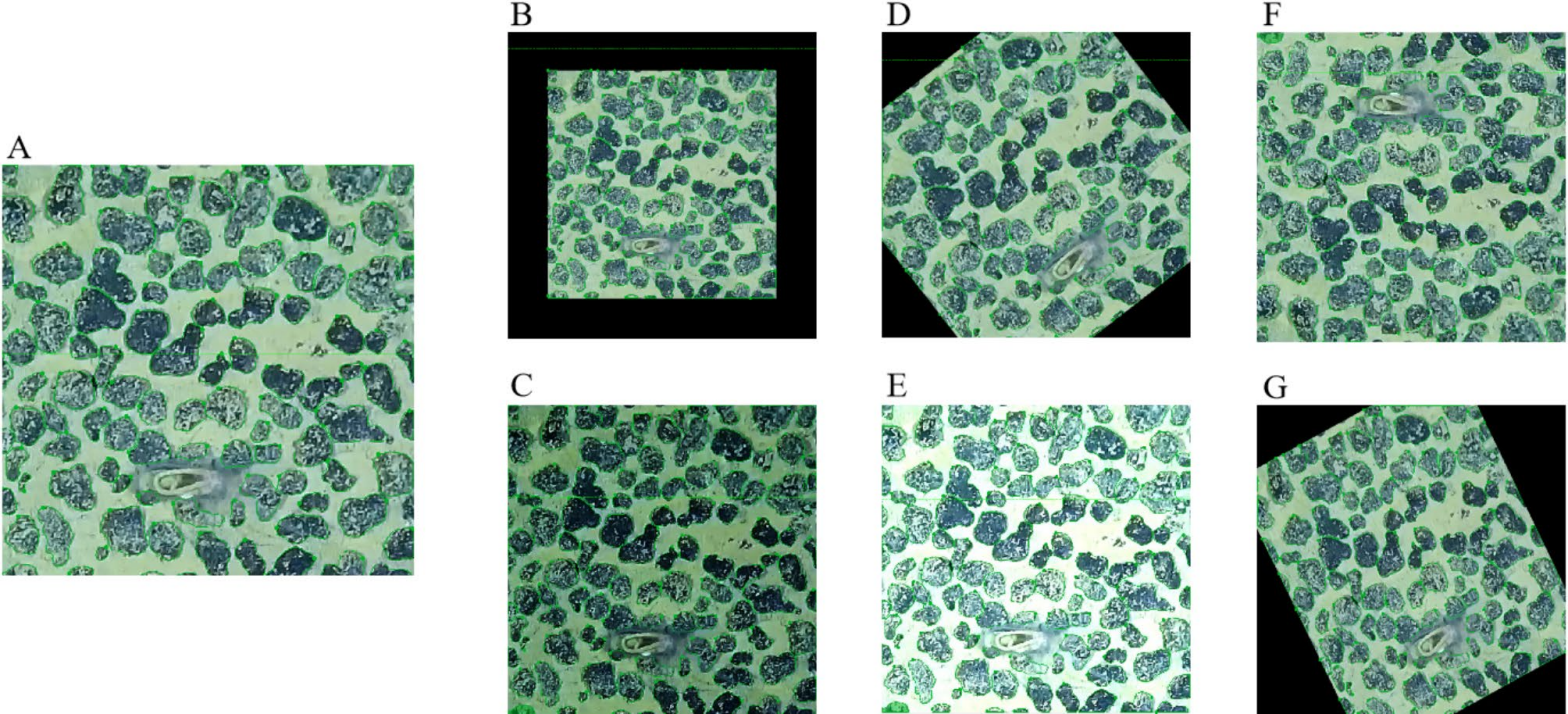
(**A**) the annotated image. Augmented images: (**B**) downscale image; (**C**) contrast image; (**D**) rotate image; (**E**) brightness image; (**F**) vertical flip image; (**G**) shear image.

Figure 10 shows an annotated image (Fig. 10A) and augmented images (Fig. 10B-G). For semi-automatic annotation of the dataset, the Segment Anything Model (SAM) from the open-source software X-Anylabeling was employed. This model was used to distinguish polymetallic nodules from other elements in each image, creating a comprehensive annotated dataset. To enhance the training set and improve the model’s generalization and robustness, data augmentation techniques were applied. Using the Augmentor library, a Python-based image augmentation tool, the images and their annotations were augmented through the following methods: (1) Random downscale: Proportional reduction of image size to simulate imaging of smaller nodules. (2) Random flip and rotate: Alterations to image orientation to replicate various underwater camera angles. (3) Brightness adjustment: Modification of image brightness to simulate different lighting conditions. (4) Random shear: Shearing images to mimic different scales and viewpoints during capture. (5) Contrast adjustment: Random changes in image contrast within a 10–200% range to emulate varying capture scenarios.

With these techniques, the final dataset for polymetallic nodule segmentation was expanded to include 342 fully annotated images (512 × 512 pixels) and 44,071 annotated nodule instances.

### Model training and testing

**Fig. 11:**
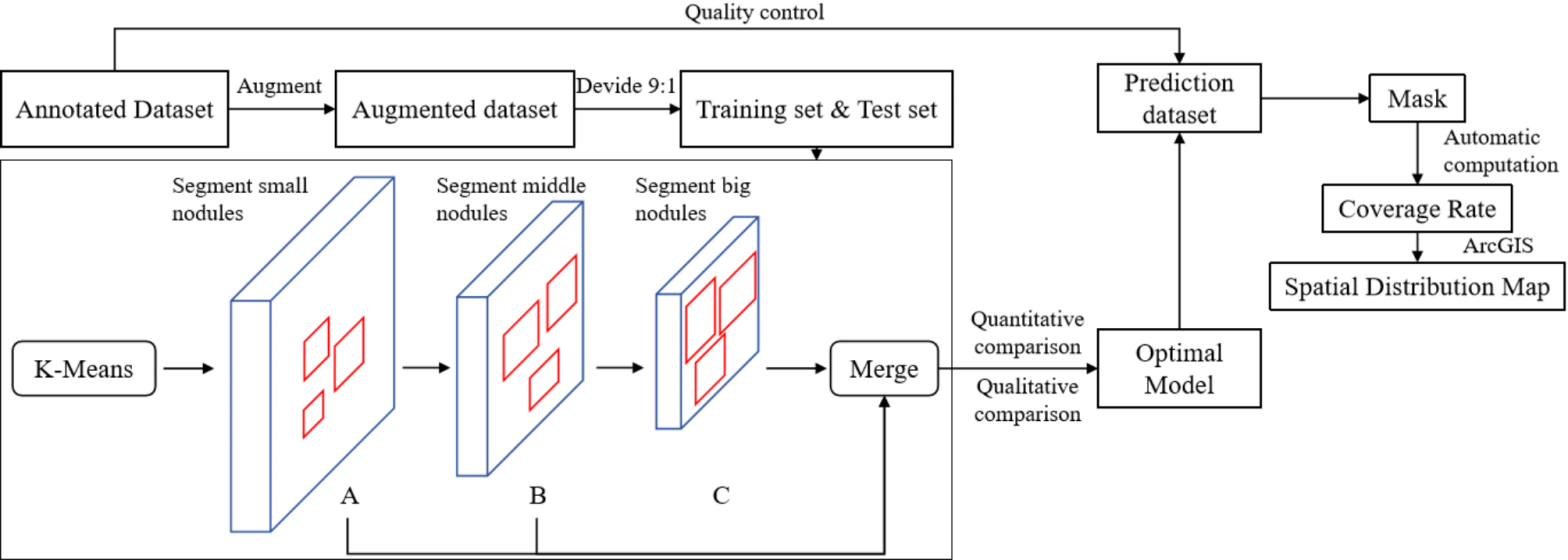
The experimental workflow for polymetallic nodule detection and segmentation using YOLOv7, YOLOv7-MobileNetV3, YOLOv7-DSC, and YOLOv7-PMN.

Figure 11 presents the experimental workflow for polymetallic nodule detection and segmentation models. These models include: YOLOv7 model and its variants—YOLOv7-MobileNetV3, YOLOv7-DSC, and YOLOv7-PMN—detailed in the “Model Improvements” section. The procedure for processing and detecting polymetallic nodule images is outlined as follows:

1. Inputting the above annotated dataset.
2. The dataset is augmented to replicate variations in camera angle, height, and lighting, producing a more robust dataset.
3. The augmented dataset is split into training and testing subsets in a 9:1 ratio, which are then used to train the four network models separately.
4. During the initial phase of model training, the K-Means algorithm is applied for label bounding box clustering, resulting in 3 groups of initial anchor boxes with 3 pairs in each group, totaling 9 pairs:[9,8], [16,15], [19,24], [27,20],[27,29], [37,30], [33,40], [44,43], [62,58]. These anchor box sizes are dynamically adjusted throughout the training process. The 512 × 512-pixel images undergo convolution to produce feature maps at three scales: Feature Map A (64 × 64 pixels): Resulting from 8× downsampling of the nodule image. Feature Map B (32 × 32 pixels): Resulting from 16× downsampling of the original nodule image. Feature Map C (16 × 16 pixels): Resulting from 32× downsampling of the original nodule image. The model extracts feature information relevant to small, medium, and large nodules from these three feature maps and merges them.
5. After completion of training, the models are evaluated using performance metrics to compare their results. The actual segmentation performance is assessed with images from both the training and prediction sets to identify the most effective model.

### Running environment

The computational environment for this model was set up with Python version 3.11.0 and utilized PyTorch version 2.1.0 + cu118 as the deep learning framework. The training was conducted on a server equipped with 2 Graphics Processing Units (GPUs) named NVIDIA GeForce RTX 4090 each featuring 24,564 MiB of video memory. Both GPUs were actively employed throughout the training process. The Central Processing Unit (CPU) used was a 13th Generation Intel (R) Core (TM) i9-13900 K, operating at a frequency of 3.00 GHz.

For optimization, the Adam optimizer was chosen with an initial learning rate of 0.01. The training regimen included 100 epochs, with a batch size of 16 images per epoch.

### Model results

#### Comparison of image segmentation on the prediction dataset

**Fig. 12:**
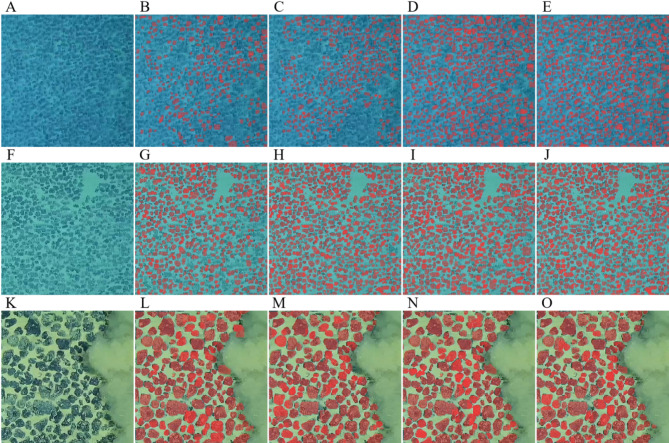
Model detection results in different scenarios. Scenarios: (**A-E**), far-distance images; (**F-J**), mid-distance images; (**K-O**), near-distance images. (**A**), (**F**), (**K**), original images; (**B**), (**G**), (**L**), YOLOv7 detection results; (**C**), (**H**), (**M**), YOLOv7-MobileNetV3 detection results; (**D**), (**I**), (**N**), YOLOv7-DSC detection results; (**E**), (**J**), (**O**), YOLOv7-PMN detection results.

Figure 12A-O provides a comparison of image segmentation results on the prediction dataset. We tested the YOLOv7, YOLOv7-MobileNetV3, YOLOv7-DSC, and YOLOv7-PMN models on Near-distance, Mid-distance, and Far-distance images from the prediction set. The results shown in Fig. 12 reveal that the YOLOv7 has the highest rate of missed detections, whereas YOLOv7-PMN achieves the lowest rate. In Near-distance images (Fig. 12K-O), all models perform well in identifying and segmenting medium and large-sized nodules, with few missed detections or boundary inaccuracies. When it comes to small nodules in Mid-distance images (Fig. 12F-J), the YOLOv7 model performs the worst. YOLOv7-DSC and YOLOv7-MobileNetV3 produce similar results, but YOLOv7-PMN stands out with the best performance. For Far-distance images (Fig. 12A-E), YOLOv7-PMN provides the most accurate detection and segmentation results (Fig. 12E).

#### Model evaluation indices

This study evaluates model performance using several metrics: recall rate, precision rate, model parameters, best weight memory, and inference speed. These metrics are defined as follows:

Recall Rate: This metric reflects the proportion of actual nodules that are correctly detected by the model. It represents the ratio of true positive detections to the total number of true nodules. A high recall rate indicates that the model effectively identifies most of the actual nodules.

Precision Rate: This metric is defined as the ratio of correctly identified positive nodules to the total number of nodules predicted as positive. A higher precision rate indicates that the model is less likely to label a negative nodule as positive.

Model parameters refer to the total number of trainable parameters within the neural network. A larger number of parameters generally signifies a more complex model that can capture intricate features. However, this complexity can lead to increased training times and higher memory usage, and an excessive number of parameters may also result in overfitting.

Best weight memory pertains to the amount of memory needed to store the optimal weight parameters of the neural network. These weights are usually stored as floating-point numbers, with each number consuming a specific amount of memory. The memory size for storing weights depends on the model’s architecture, the quantity of parameters, and the precision of the weights. High-weight memory usage can impact the model’s performance on devices with limited memory, lengthen training periods, slow down model loading, and increase deployment costs.

Inference speed denotes the time required for the model to process input data and generate predictions. This metric is crucial for applications demanding quick responses and reflects the model’s efficiency in practical scenarios.

**Table 1:** Comparison of model evaluation indices.

	YOLOv7	YOLOv7-MobileNetV3	YOLOv7-DSC	YOLOv7-PMN
Recall Rate	94%	*97%*	*97%*	*97%*
Precision Rate	91.12%	91.52%	92.94%	*95.62%*
Model Parameters	37,866,092	24,650,220	20,048,045	*14,472,068*
Best Weight Memory	76.2 MB	49.8 MB	40.9 MB	*29.6 MB*
Inference Speed	40.65 FPS	62.50 FPS	55.86 FPS	*65.79 FPS*

Significant values are in italics.

Table 1 illustrates that the YOLOv7 model has the lowest recall rate, the lowest precision rate, the highest number of parameters, the greatest memory usage for storing optimal weights, and the slowest inference speed. When utilizing the MobileNetV3-Small feature extraction structure as its backbone, the YOLOv7-MobileNetV3 model achieves a high recall rate of 97% and a precision rate of 91.52%. It also reduces the total number of parameters by 13,215,872 and decreases both the memory used for weight storage and the inference time significantly. The YOLOv7-DSC model, which incorporates depth-wise separable convolution, further optimizes the improvement of precision rate and the reduction of model parameters and memory usage compared to the standard YOLOv7. The YOLOv7-PMN model stands out with around 14.47 million parameters, which is a reduction of 23.39 million from YOLOv7. It uses only 29.6 MB of memory for weights and reaches an inference speed of 65.79 FPS, which is notably faster than typical camera capture rates. Its high recall rate of 97% and precision rate of 95.62% indicates strong application accuracy.

In summary, the YOLOv7-PMN model excels in performance metrics, featuring a high recall rate, minimal parameters, low weight memory usage, and rapid inference speed.

#### Comparative evaluation of model coverage rate

To evaluate the precision of nodule boundary segmentation, this study calculates the nodule coverage rate based on annotated images as the reference (labeled coverage rate) and compares it with the coverage rate detected by the model (detected coverage rate). The deviation rate (δ), which measures how much the detected coverage rate deviates from the labeled coverage rate, is computed using the formula:

4 $$\delta = \left| {{\text{detected value }} - {\text{ labeled value}}} \right|/{\text{ labeled value}}$$

**Fig. 13:**
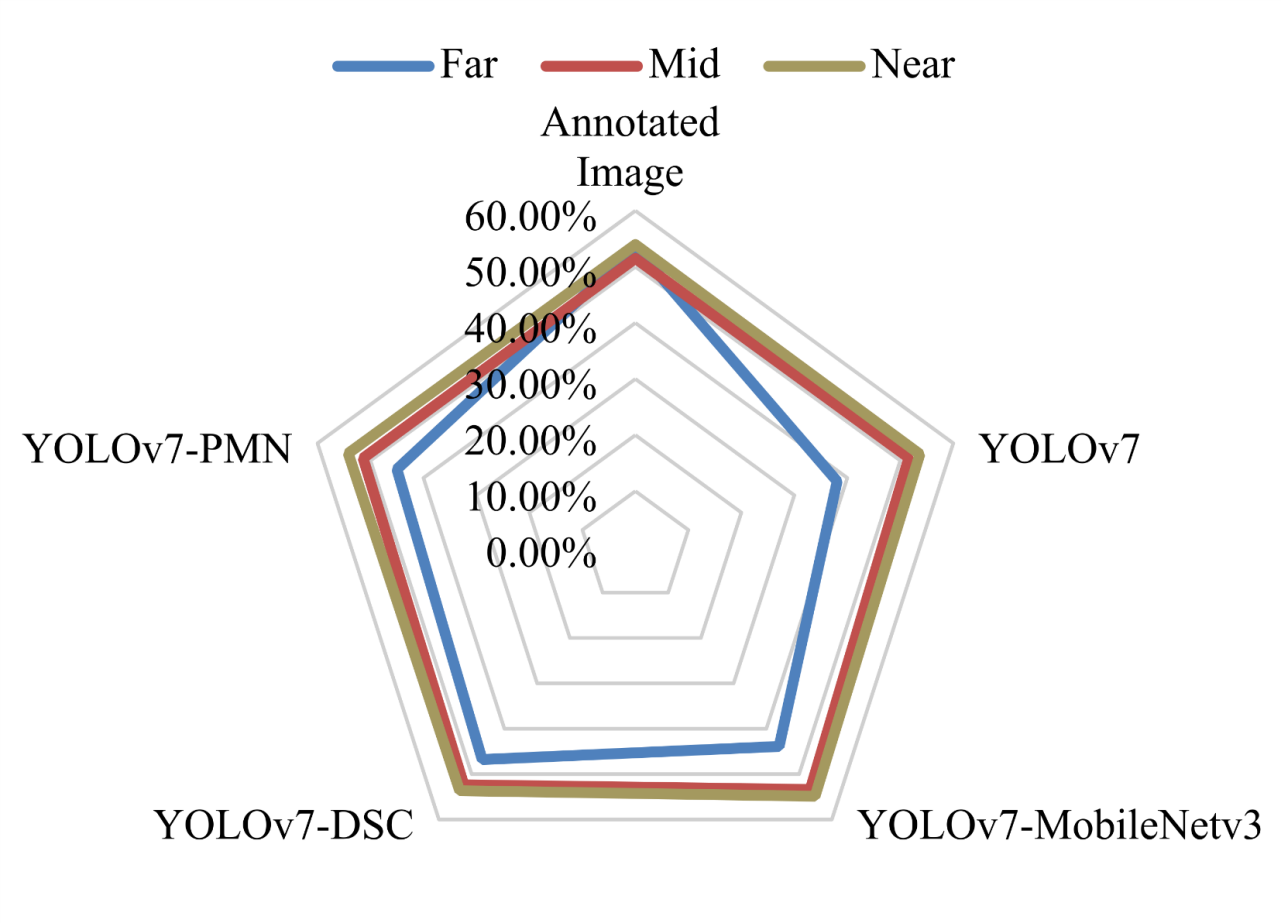
Model-detected CR vs. Annotated CR.

Far-distance (Fig. 9A), mid-distance (Fig. 9D), and near-distance image (Fig. 9G) exhibit labeled coverage rates of 53.45%, 51.51%, and 53.97%, respectively. The coverage rates detected by the four models for these image categories are recorded. For instance, the YOLOv7 model achieved detected coverage rates of 38.08%, 51.61%, and 53.69% for Far-distance, Mid-distance, and Near-distance images, respectively, as shown in Table 2. Using these detected values and the labeled values, deviation rates (δ_1_ for Far-distance, δ_2_ for Mid-distance, and δ_3_ for Near-distance) are calculated. Table 2 reveals that the YOLOv7-PMN model consistently shows lower deviation rates across all image distances. The radar chart in Fig. 13 provides a visual representation of the deviation in coverage rates for nodule images captured at different distances by each model.

**Table 2:** Comparison of annotated and detected image coverage rates.

	Far-distance image		Mid-distance image		Near-distance images	
	CR	δ_1_	CR	δ_2_	CR	δ_3_
Annotated Image	53.45%	-	51.51%	-	53.97%	-
YOLOv7	38.08%	0.2876	51.61%	0.0019	53.69%	0.0052
YOLOv7-MobileNetv3	43.90%	0.1787	53.39%	0.0365	54.87%	0.0167
YOLOv7-DSC	46.79%	0.1246	52.37%	0.0167	53.66%	0.0057
YOLOv7-PMN	45.01%	0.1579	51.40%	0.0021	54.20%	0.0043

Note: CR is the Coverage Rate; δ_1_, δ_2_, and δ_3_ represent the deviation rates for far-distance, mid-distance, and near-distance images, respectively.

Through an extensive evaluation of model training metrics, comparisons of coverage in the training set images, and assessments of segmentation performance in the prediction set images, we concluded that YOLOv7-PMN emerged as the most effective model for this study.

### Application of the YOLOv7-PMN

Once the optimal model was built, it was employed to analyze survey lines and compute nodule coverage rates through video examination. During this prediction phase, the model utilizes the features it has learned to predict nodule pixels across various scale feature maps for a given image. When a sub-region contains nodule pixels, the model produces a segmented area. These segmented areas are then combined to create a binary segmentation result, which distinguishes nodules from the background, thus generating mask images for all the input images. The YOLOv7-PMN model subsequently calculates the nodule coverage rate for these mask images using a coverage rate formula. Finally, ArcGIS was used to spatially visualize the coverage rate data based on the estimated nodule coverage rates and positioning information.

**Fig. 14:**
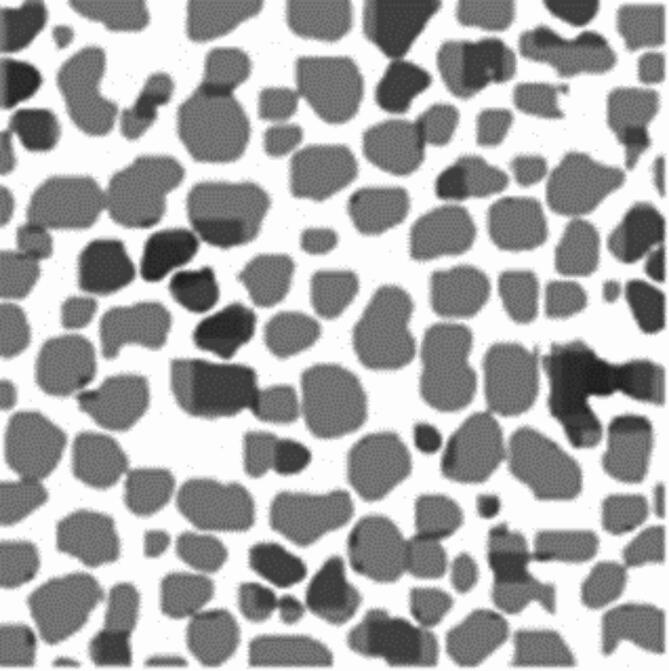
Semantic segmentation results of nodule images. In these images, white pixels represent non-nodule class pixels and gray pixels represent nodule class pixels.

Figure 14 displays the results of the semantic segmentation of nodule images. In these visualizations, white pixels denote areas without polymetallic nodules, while gray pixels highlight the areas containing polymetallic nodules.

The coverage rate of polymetallic nodules in an image can be obtained using the following expression:

5 $$\:\text{F}=\frac{{\text{N}}_{\text{w}\text{i}\text{d}\text{t}\text{h}}\ast\:{\text{N}}_{\text{h}\text{e}\text{i}\text{g}\text{h}\text{t}}-{W}_{i}}{{\text{N}}_{\text{w}\text{i}\text{d}\text{t}\text{h}}\ast\:{\text{N}}_{\text{h}\text{e}\text{i}\text{g}\text{h}\text{t}}}$$

where $\:{\text{N}}_{\text{w}\text{i}\text{d}\text{t}\text{h}}$ and $\:{\text{N}}_{\text{h}\text{e}\text{i}\text{g}\text{h}\text{t}}$ represent the width and height of the mask in pixels, respectively, and $\:{\text{W}}_{\text{i}}$ denotes the number of non-nodule pixels within the mask. In Fig. 14, the calculated coverage rate of polymetallic nodules is 55.52%.

**Fig. 15:**
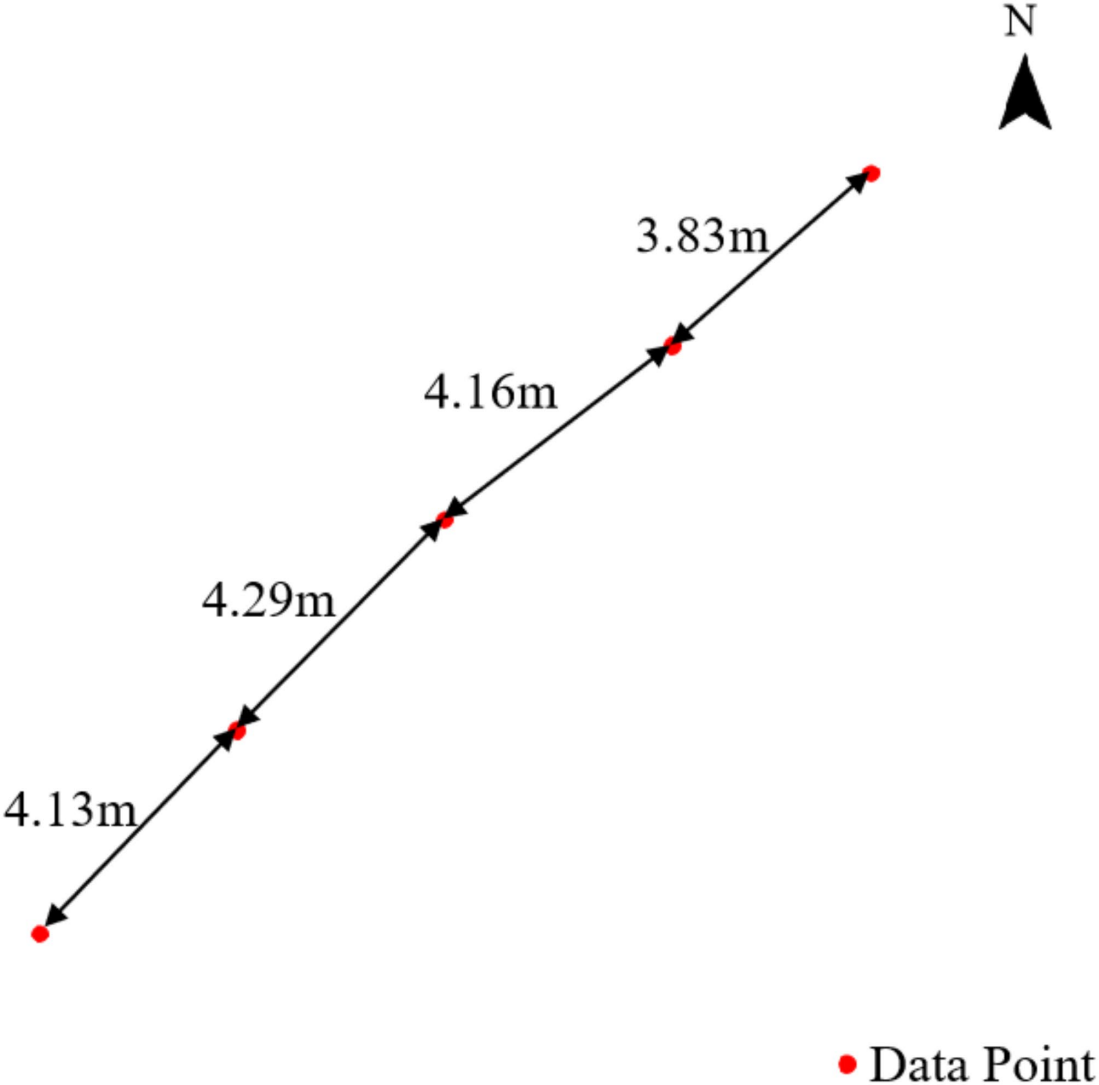
Spatial Distribution of adjacent data points.

**Fig. 16:**
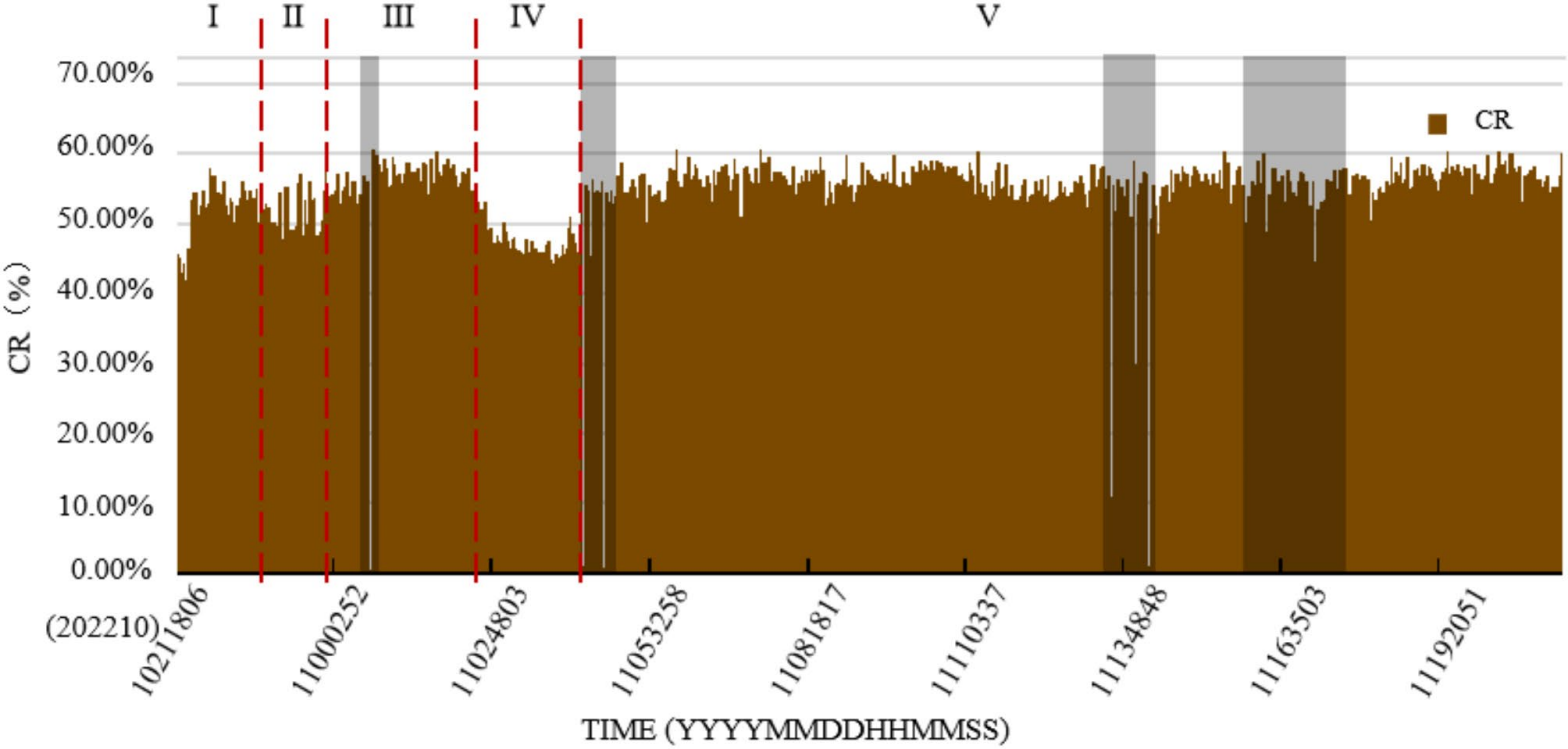
Coverage calculations based on the YOLOv7-PMN model across the entire video survey line.

To check the performance of the YOLOv7-PMN model, it was deployed across the entire survey line. The sampling frequency for the images to be recognized for computation was set to 250 frames per 10 s, resulting in an average spatial interval of about 4 m between acquired images (Fig. 15). A total of 8,866 images were collected, though 82 images were excluded due to quality issues. The results indicate that the model achieves an inference speed of 65 frames per second, enabling the calculation of polymetallic nodule coverage for the entire measurement line, consisting of 8,784 images, in approximately 2.3 min. Figure 16 outlines the variation in coverage rates along the video survey line, categorized into five distinct segments:

I. Nodule coverage rate around 53%;
II. Nodule coverage rate declining to about 50%;
III. Nodule coverage rate rising to 55%;
IV. Nodule coverage rate dropping sharply to 45%;
V. Coverage rate varying between 50% and 57%.

**Fig. 17:**
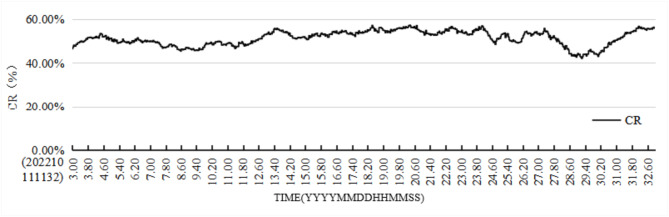
Continuity distribution of polymetallic nodule coverage based on video.

To check the continuity and smoothness of the results of the polymetallic nodule coverage calculations using the model, a 30-second video clip was selected from a period when the equipment-carrying platform was operating stably. Images were sampled at a rate of 1 frame every 40 milliseconds. In total, 750 frames were processed. The model accurately detected and segmented nearly all nodules in these frames and calculated the nodule coverage rate for each. Figure 17 presents these results, demonstrating that the calculated distribution of polymetallic nodule coverage aligns with the general distribution.

## Discussion

Acquiring near-seafloor imagery is currently the most effective method for detecting polymetallic nodules. Nonetheless, this process faces challenges due to the harsh deep-sea environment and the limitations of data collection equipment. The absence of natural light on the seafloor means that artificial lighting must be used, but this is significantly affected by the water medium. Longer wavelengths, such as red and orange, are attenuated quickly as the distance from the light source increases, whereas blue and green wavelengths penetrate deeper. As detailed in the section on Image Data, images taken from a greater distance from the seafloor often exhibit a blue-green hue, while closer images have darker yellow tones. Additionally, suspended particles in the water scatter light, diminishing intensity and causing image blurriness. This means that nodule boundaries become less distinct with increased distance from the imaging equipment, while proximity results in clearer images. Furthermore, safety protocols necessitate that platforms maintain a certain distance from the seafloor, limiting the collection of stable Near-distance data and leading to images of smaller nodules. Variations in movement speed can also introduce motion blur, complicating nodule detection and coverage rate assessment. The polymetallic nodules themselves are small, morphologically diverse, irregular, and unevenly distributed. This variability results in complex image characteristics, including diverse target features, multi-scale representations, unstable image quality, and significant environmental influences like occlusions and concealment. To effectively detect nodules in such conditions, the model must maximize the detection of multi-scale targets while accommodating various unstable imaging scenarios. Additionally, with the increasing need for real-time applications in deep-sea exploration and mining, achieving a balance between detection accuracy and processing speed is crucial. Thus, a model capable of processing diverse and multi-scale images efficiently, with minimal computation time and memory usage, is essential for detailed exploration of deep-sea polymetallic nodules. To address these challenges, this study developed a lightweight YOLOv7-PMN semantic segmentation model. This model has been optimized for improved detection speed, reduced computational load, enhanced accuracy, better detection of small targets, and increased generalization ability.

To enhance target detection and segmentation speed, the new model incorporates the MobileNetV3-Small feature extraction structure. This structure utilizes 11 relatively straightforward and linear bneck modules, which streamline the feature extraction process and improve detection and segmentation speed. The segmentation prediction dataset used in the present study contains 8,866 images, including 1,380,792 nodule instances. Table 1 reveals that models with the MobileNetV3-Small feature extraction, such as YOLOv7-MobileNetV3 and YOLOv7-PMN, achieve inference speeds of 62.50 FPS and 65.79 FPS, respectively. In contrast, models without this feature extraction structure, like YOLOv7 and YOLOv7-DSC, exhibit lower inference speeds of 40.65 FPS and 55.86 FPS. The UNET model got an inference speed of 47.85 FPS when tested on the same dataset as the proposed model. In contrast, the YOLO-PMN model, enhanced with the DSC, reached the highest inference speed of 65.79 FPS among all tested models. This observation demonstrates that both MobileNetV3 and DSC contributed significantly to improving inference speed. The effectiveness of the MobileNetV3-Small structure in accelerating model inference speed is particularly noteworthy. MobileNet-based architectures are widely recognized for their ability to balance computational efficiency and accuracy, making them an appropriate choice for edge computing and real-time applications^40,43^. Recent studies, such as Bauravindah et al.^54^ highlighted the superior inference speed of MobileNet (439.56 FPS) compared to lightweight networks like ShuffleNet (188.68 FPS) and EfficientNet (182.15 FPS) on BOSSbase-1.01 steganalysis dataset with 2,000 images (512 × 512 pixels). Additionally, in real-time deep-sea applications, where the rapid analysis of seafloor imagery is critical, achieving inference speeds exceeding 60 FPS represents a significant improvement. For instance, Tomczak et al.^55^ proposed a UNet model specifically trained to process 30,000 offshore nodule images in under 10 h (0.83 FPS). In comparison, the proposed solution demonstrates a clear advantage in handling real-time detection and segmentation tasks in challenging underwater environments. Faster processing is crucial for real-time applications in deep-sea environments, where rapid analysis of seafloor imagery is often required. The ability to process images at speeds exceeding 60 FPS represents a significant improvement in handling large datasets efficiently, enabling more timely and detailed assessments of polymetallic nodule distributions.

Regarding model weight memory usage reduction, the new model also introduces depth-wise separable convolution. As detailed in section Depth-wise Separable Convolution (“YOLOv7-DSC”), this technique breaks down standard convolution into two steps: depth-wise convolution and point-wise convolution, which substantially reduces computational complexity and parameter count. Table 1 shows this reduction: (1) YOLOv7-DSC (20,048,045 parameters), which uses depth-wise separable convolution, reduces the number of parameters by 17,818,047 compared to YOLOv7 (37,866,092 parameters), leading to a decrease of 35.3 MB in weight memory; (2) YOLOv7-PMN (14,472,068 parameters), which applies depth-wise separable convolution in its head network, reduces parameters by 10,178,152 compared to YOLOv7-MobileNetV3 (24,650,220 parameters), resulting in a 20.2 MB reduction in weight memory. In the field of image segmentation, models like DeepLabV3+^56^ have adopted similar strategies to enhance efficiency. However, many such approaches rely on pre-trained backbones or additional processing steps, which can increase overall complexity. When compared to lightweight segmentation models such as ENet (about 370,000 parameters with a global average accuracy of 59.5% on the SUN dataset)^57^ and Fast-SCNN (about 1,110,000 parameters with an accuracy below 80% on the Cityscapes dataset)^58^, which often comprise accuracy to achieve smaller model sizes, the YOLOv7-PMN model retains high segmentation accuracy while reducing memory usage. In summary, the YOLOv7-PMN model demonstrates distinct advantages in resolving the trade-off between segmentation accuracy and memory efficiency. This is mainly attributed to its integration of DSC modules, offering competitive solutions for real-world segmentation tasks.

To enhance target detection and segmentation accuracy, the integration of the MobileNetV3-Small feature extraction structure with depth-wise separable convolution proves highly effective. The YOLOv7-PMN model incorporates 9 bneck modules with SE components in its backbone network (as detailed in the MobileNetV3 Network section). The SE module processes convolutional feature maps through global average pooling to generate descriptors for each channel. These descriptors are then fed through two fully connected layers with ReLU and Hard-sigmoid activation functions, allowing for both nonlinear and linear feature evaluation, augmentation, and selection. This process produces importance weights for each channel, which are multiplied by the original feature map to create an adjusted feature map. The SE module thus highlights the most informative channels while suppressing less relevant ones, thereby enhancing the quality and discrimination of feature representations. In parallel, depth-wise separable convolution facilitates the development of deeper networks with fewer parameters than standard convolution. This reduction in parameters improves feature extraction capabilities by focusing on essential features more effectively. The combination of these techniques effectively addresses the challenges posed by low-quality images, which can result from varying target sizes, capture distances, and environmental factors. It also enhances the delineation of nodule boundaries against the background. As shown in Table 1, models incorporating either YOLOv7-MobileNetV3, YOLOv7-DSC, or YOLOv7-PMN all achieve a high recall rate of 97%. Table 2 further reveals that YOLOv7-PMN exhibits lower detection deviation in nodule coverage rates across different scales of images. Figure 12 confirms that YOLOv7-PMN surpasses both YOLOv7-MobileNetV3 (which solely uses the MobileNetV3-Small feature extraction structure in its backbone) and YOLOv7-DSC (which relies solely on depth-wise separable convolution throughout the YOLOv7 network) in detection accuracy. These findings align with prior research. Howard et al.^40^ demonstrated that the MobileNetV3-Small model with SE components achieved 0.53 billion Multiply-Accumulate Operations (MAdds) and 68.3% mean Intersection over Union (mIOU), outperforming ESPNetv1 on the Cityscapes semantic segmentation dataset. Similarly, Chollet^59^ proposed the Xception model, which mainly utilized depth-wise separable convolutions to reduce complexity without comprising performance. The Xception model achieved approximately 80% accuracy on ImageNet with 22,855,952 parameters. By integrating advanced feature extraction techniques with computational efficiency, the YOLOv7-PMN model exhibits excellent performance, demonstrating its robustness and suitability for real-time applications.

**Fig. 18:**
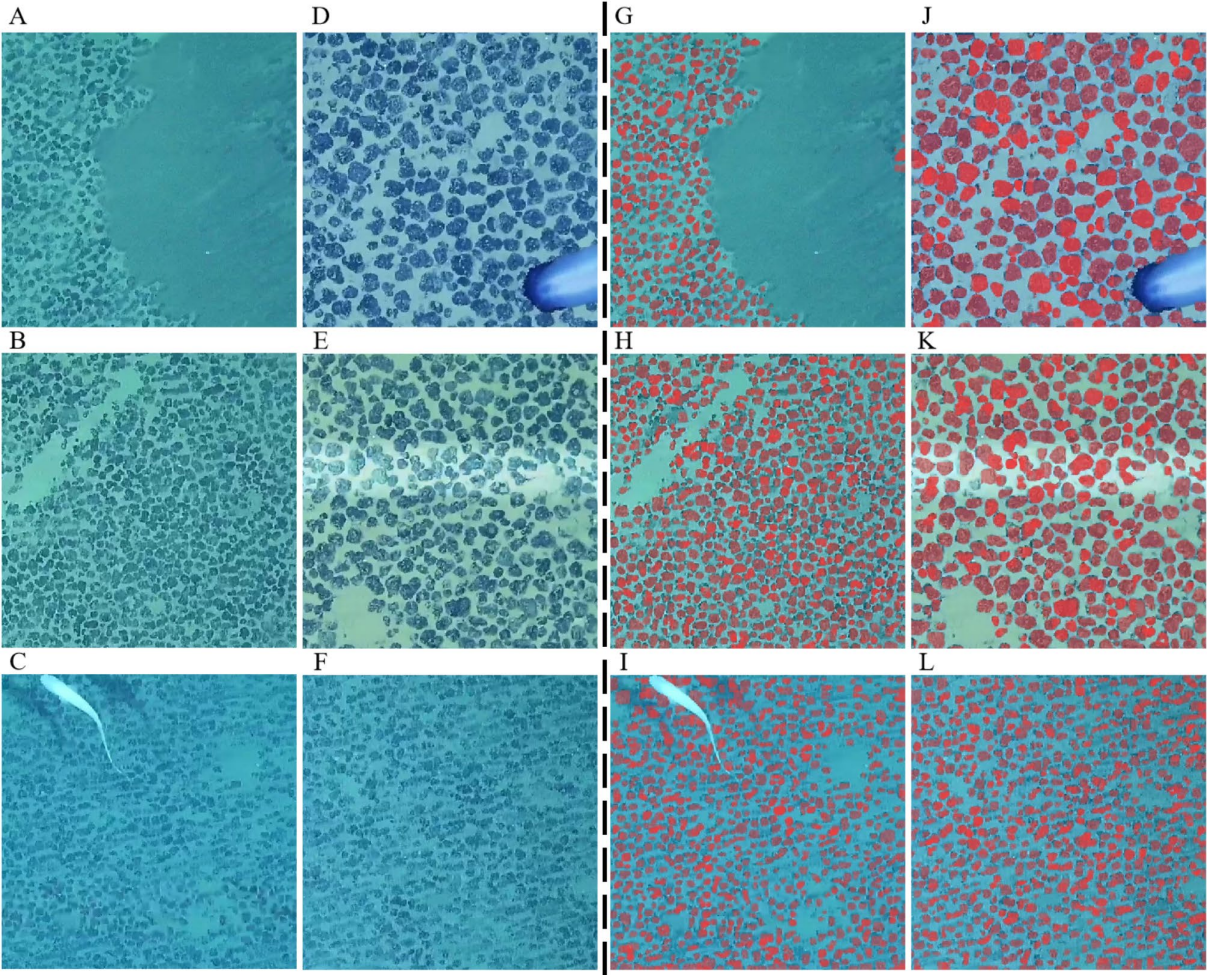
(**A-F**), prediction subset. (**G-L**), prediction results using YOLOv7-PMN.

To enhance small-sized target detection, the YOLOv7-PMN model incorporates several advanced techniques: (1) K-Means clustering: This model employs the K-Means clustering algorithm to group boundary boxes of all targets in the training set. This clustering results in the generation of initial anchor boxes based on the size and shape distributions observed in the data. Similar to this approach, the YOLOv4 model improved anchor box suitability for diverse object scales and achieved an average precision of 43.5% for the MS COCO dataset at a speed of 65 FPS on Tesla V100^60^. (2) Dynamic anchor box adjustment: During training, the model continuously evaluates detection performance for targets of varying sizes. It dynamically adjusts the sizes and ratios of the anchor boxes according to performance metrics to optimize detection accuracy. This adaptive strategy, similar to the FreeAnchor model^61^, enhances detection accuracy, particularly for small-sized targets. (3) Multi-scale anchor boxes: This model creates anchor boxes of different sizes for feature maps at various scales, ensuring that appropriate anchor boxes are available for small, medium, and large targets. This module aligns with multi-scale feature integration in models like RetinaNet^62^ which achieved 39.1% average precision on COCO test set. (4) SE mechanism: The SE attention mechanism is utilized to extract both spatial and channel features. This helps in emphasizing important features and suppressing less relevant ones, enhancing the model’s ability to detect and segment nodules of different sizes. These techniques collectively improve the YOLOv7-PMN model’s capability to detect and segment polymetallic nodules of various sizes across near-distance, mid-distance, and far-distance images. Table 2 indicates that YOLOv7-PMN has the lowest detection deviation rate for near-distance and mid-distance images and performs second-best for far-distance images. In Fig. 12, YOLOv7-PMN shows the fewest missed detections and boundary misdetections across images taken at different distances. Figure 18A-F shows the prediction subset, while Fig. 18G-L presents the prediction results using YOLOv7-PMN. Figure 18A-C, F highlights YOLOv7-PMN’s effectiveness in detecting small nodules of various shapes and sizes in images. This enhanced performance in small-sized target detection is vital for polymetallic nodule exploration, as accurately identifying smaller nodules can greatly impact resource estimation and environmental assessments. The model’s adaptability to different imaging conditions and target sizes represents a significant advancement in deep-sea nodule detection and segmentation.

Regarding model generalization capability, the YOLOv7-PMN model has demonstrated notable performance. As shown in Fig. 18, the prediction set images (Fig. 18A-F) include organisms (Fig. 18C, D) with various species, colors, shapes, and sizes, differing significantly from those in the training set (Fig. 9). Additionally, these images contain suspended matter (Fig. 18A) with diverse shapes, areas, and concentrations, as well as sedimentation voids (Fig. 18B) of varying forms and sizes. Figure 18E features bright bands caused by camera malfunctions. Furthermore, the prediction set includes a larger number of mid- and far-distance images compared to the training set. The YOLOv7-PMN model’s detection and segmentation results for Fig. 18A-F are displayed in Fig. 18G-L, respectively. The model successfully detected nearly all nodules, with predicted contours closely matching the actual contours. For instance, in Fig. 18D, small nodules partially obscured by blue organisms were also detected. Despite increased blur in Fig. 18C, F compared to the training set, the model effectively identified and segmented the nodules. In Fig. 18C, where a suspended aquatic organism (white fish) cast shadows on the nodule surface, the YOLOv7-PMN model accurately detected and segmented the nodules even within the shadowed regions. This capability underscores the model’s proficiency in distinguishing between pixels of similar colors that belong to different categories. The model’s performance in detecting nodules, even in cases lacking specific label types in the training set—such as images with significant blur or various occlusion conditions—further validates its strong generalization capabilities. These results indicate that YOLOv7-PMN is highly adaptable and effective across diverse and challenging imaging scenarios.

Currently, research into the continuous real-time evaluation of polymetallic nodule coverage changes at meter-scale spatial resolution near the seafloor is limited. Traditional box sampling methods often use spatial distances of 3.5 km or more, with each sampling covering roughly 0.25 m^2^. In contrast, imaging systems offer a more continuous high-resolution approach for monitoring polymetallic nodules on the seafloor, typically covering widths greater than 6 m. The area captured in a single image can reach up to 30 m^2^, depending on the altitude of the imaging equipment above the seafloor. The YOLOv7-PMN semantic segmentation model introduced in this study provides real-time, precise image detection, exceeding the frame rate of image capture. This model is designed to handle coverage estimation across various spatial scales based on specific application needs. For example, as shown in Fig. 17, the sampling interval allows for analyzing nodule coverage at centimeter-scale resolution. Figure 15 illustrates meter-scale variations in coverage, while Fig. 16 covers kilometer-scale changes along an entire survey line.

Despite its advancements, the YOLOv7-PMN model’s performance in detecting and estimating nodule coverage is still limited by the quality of the images. It struggles to identify nodules in very poor-quality images, such as those from a great distance from the seafloor or where lighting conditions are suboptimal. The accuracy of nodule coverage estimates is further impacted by occlusions from organisms, suspended particles, and other obstructions. To optimize the effectiveness of the model, efforts should focus on enhancing image quality screening and evaluation. The imaging platform should maintain a stable, low altitude above the seafloor and move at a moderate pace to improve image quality and reduce interference from suspended sediments. Additionally, for more accurate nodule coverage estimates, the areas covered by occluding objects should be excluded from calculations. When annotating datasets, it is advisable to separately label objects other than nodules and sediments, classifying them into occluding objects (like organisms and suspended particles) and non-occluding objects (such as sediment areas) etc.

## Conclusion

This study addresses the pressing need for continuous, real-time, and precise monitoring of polymetallic nodule coverage in complex seafloor environments. It takes into account the challenges posed by multi-scale and multi-feature nodules and the necessity for low-memory model deployment in nodule exploration. Using the Python programming environment, we developed the YOLOv7-PMN model—a streamlined deep-sea polymetallic nodule coverage estimation tool. The YOLOv7-PMN model utilizes the connections between its backbone network and the head network, as well as the intra-module connections within the head network, to facilitate the efficient transfer of multi-scale feature information. This design enables effective detection and segmentation of objects of different sizes within an image. The model’s backbone features bneck modules with SE attention mechanisms, and its head network incorporates depth-wise separable convolutions. After the segmentation operation, the coverage estimation method was added to the model. This integration facilitates automatic nodule coverage calculations within the model. By correlating temporal nodule coverage data with positioning information, we achieved spatial visualization of nodule distribution, which aids in analyzing spatial continuity. The YOLOv7-PMN model achieved a 97% recall rate in nodule detection, surpassing the YOLOv7 model by 3%. Its average inference speed is approximately 8.69 FPS on the CPU and 65.79 FPS on the GPU, significantly outpacing YOLOv7’s GPU average inference speed of 40.65 FPS. The model’s parameter count was reduced by 61.78%, and its weight memory usage is 29.6 MB—just 38.85% of that used by the YOLOv7 model. These enhancements meet the requirements for efficient, low-memory deployment. Additionally, YOLOv7-PMN exhibits improved capabilities in detecting and segmenting small-scale targets and demonstrates superior generalization performance. Application of the YOLOv7-PMN model to near-seafloor nodule images shows its ability to swiftly and accurately detect, segment, and calculate nodule coverage rates. This research would provide valuable support for understanding the spatial distribution of nodule mineral resources as well as for detailed exploration and future mining.

## Data Availability

The dataset used and analyzed in this study is available from the corresponding author S.Y. upon reasonable request.
